# 4D (x-y-z-t) imaging of thick biological samples by means of Two-Photon inverted Selective Plane Illumination Microscopy (2PE-iSPIM)

**DOI:** 10.1038/srep23923

**Published:** 2016-04-01

**Authors:** Zeno Lavagnino, Giuseppe Sancataldo, Marta d’Amora, Philipp Follert, Davide De Pietri Tonelli, Alberto Diaspro, Francesca Cella Zanacchi

**Affiliations:** 1 Department of Nanophysics, Istituto Italiano di Tecnologia, Genova, Italy; 2 DIBRIS, Università di Genova, Genova, Italy; 3 Department of Neuroscience and Brain Technologies, Istituto Italiano di Tecnologia, Genova, Italy; 4 Nikon imaging Center, Istituto Italiano di Tecnologia, Genova, Italy; 5 Department of Physics, Università di Genova, Genova, Italy

## Abstract

In the last decade light sheet fluorescence microscopy techniques, such as selective plane illumination microscopy (SPIM), has become a well established method for developmental biology. However, conventional SPIM architectures hardly permit imaging of certain tissues since the common sample mounting procedure, based on gel embedding, could interfere with the sample morphology. In this work we propose an inverted selective plane microscopy system (iSPIM), based on non-linear excitation, suitable for 3D tissue imaging. First, the iSPIM architecture provides flexibility on the sample mounting, getting rid of the gel-based mounting typical of conventional SPIM, permitting 3D imaging of hippocampal slices from mouse brain. Moreover, all the advantages brought by two photon excitation (2PE) in terms of reduction of scattering effects and contrast improvement are exploited, demonstrating an improved image quality and contrast compared to single photon excitation. The system proposed represents an optimal platform for tissue imaging and it smooths the way to the applicability of light sheet microscopy to a wider range of samples including those that have to be mounted on non-transparent surfaces.

Light sheet microscopy has been proven to be an optimal imaging tool in developmental biology, and more generally for the investigation of thick biological samples^1^,^2^.

Light sheet microscopy basically relies on the use of two decoupled optical paths for illumination and detection: a widefield based detection path for fast imaging and an orthogonally oriented illumination one, responsible for confining the illumination within a thin planar region in the focal region.

Regardless of how the illumination sheet is created, either using a cylindrical lens^1^ or rapidly scanning a gaussian beam^3^, the light sheet provides intrinsic optical sectioning and three dimensional imaging capabilities with significant background rejection.

This approach, preventing the illumination of the whole sample volume, is particularly convenient for imaging of thick biological samples, such as embryos and whole brain^4^,^5^,^6^, since the imaging performances will strongly benefit of both the background reduction and the reduced phototoxicity.

The unique features of light sheet fluorescence microscopy increase the penetration depth in the specimen and reduce the overall photobleaching.

However, conventional implementations of selective plane illumination microscopes (SPIM)^7^ require a suitable sample mounting, such as embedding the sample in a gel cylinder, thus preventing the possibility of imaging a huge variety of samples and tissues to be hold in common holders, such as glass slides or petri-dishes, in order to preserve their structure.

Within this scenario the development of different architectures for light sheet microscopy, able to access the sample in a different geometry such as the ones based on up-right or single lens configurations, represents a key aspect to improve the flexibility and the application range of light sheet based microscopy.

Highly Inclined and Laminated Optical sheet microscopy (HILO) and Oblique Plane Microscopy for example, take advantage of a single high-NA objective to illuminate the sample at a given angle^8^,^9^.

An alternative approach has been implemented by using an AFM cantilever as a mirror close to the sample, deflecting the illumination from a vertically oriented objective to generate a horizontal illumination plane^10^. Still, these techniques are particularly suited for high resolution and super-resolution imaging of small regions within the sample, due to the limitation on the reachable field of view.

Recently a new development, based on a single objective, has also been demonstrated to be an optimal tool to perform super-resolution within a light sheet based architecture^11^.

A promising light sheet based approach, able to combine all the advantages of light-sheet microscopy and the compatibility with standard sample geometry, is represented by inverted Selective Plane Illumination Microscopy (iSPIM)^12^. Such architecture doesn’t require agar-based sample mounting, widening the range of application of light sheet microscopy like imaging of brain slices, which are usually incompatible with gel embedding. A similar approach has also been showed to be suitable for live-cell imaging^13^.

Even if the range of application of light sheet based imaging can be improved by eliminating the agar-based embedding process, the orthogonality between excitation and detection lenses still prevents the use of the highest available numerical aperture (NA) objectives, thus limiting the axial resolution to several micrometers. The dual-view inverted SPIM approach (diSPIM) allows spatially isotropic resolution by switching illumination and detection between the objectives in an alternate fashion^14^.

Unfortunately, this approach requires two cameras and two identical objectives, which limits the tunability of the dimension of the light sheet together with the detectable field of view. Moreover, the dual view approach doubles the acquisition time and increases dramatically the amount of data collected.

Other solutions to improve the axial resolution have recently been demonstrated exploiting different excitation regimes, such as Bessel or Airy beams^15^,^16^,^17^, showing an impressive axial resolution improvement, unfortunately accompanied by stronger photobleaching effects introduced by the out of focus illumination. Especially when imaging large biological samples, aberrations deriving from light-matter interactions can significantly affect the image formation process^18^,^19^ and, in the case of light-sheet microscopy, may lead to an increased “striping effect” due to a distortion of the excitation volume.

In order to reduce aberrations caused by scattering and to improve the image quality several light-sheet-based multiview approaches have also been established^20^,^21^,^22^.

Furthermore, since scattering is inversely proportional to the incident wavelength, the use of red-shifted wavelengths required for two-photon excitation (2PE) allows for the reduction of scattering-induced effects, whilst still providing intrinsic optical sectioning capabilities^23^,^24^,^25^,^26^.

Within this scenario, the combination of two-photon excitation and SPIM has recently been demonstrated^27^,^28^,^29^ and the benefits brought by nonlinear excitation have been shown, both in terms of aberrations reduction^30^ and multicolor capabilities^31^. These advantages play a relevant role while imaging biological tissues, since particular attention has to be addressed to the imaging depth and the reachable image contrast.

However, all these non-linear techniques remain implemented in conventional light sheet based architecture and they can hardly be applied to imaging samples and tissues which are usually incompatible with the gel-based mounting required for SPIM.

In this work we present a light sheet imaging system for 2PE based on an inverted SPIM architecture (2PE-iSPIM), able to enhance the image quality, the signal to noise ratio (SNR) and the imaging depth capabilities in thick biological samples.

Such an optical system represents a suitable imaging solution both for whole organisms and tissues, smoothing the way for imaging samples incompatible with agarose gel embedding, such as hippocampal slices from mouse brain. The high accessibility of the sample from different directions provided by the optical system allows for easy buffer exchange and sample manipulation, thus permitting long term experiments on live animals such as Zebrafish embryos. Additionally, this system geometry allows for eventual coupling with heating systems, such as objective heaters, and custom made incubator chambers depending upon the sample requirements (e.g. living tissues that require oxygen and CO2).

Moreover, the removal of the classical gel embedding, peculiar to the conventional SPIM approaches, makes two-photon iSPIM an effective solution also to avoid growth defects in embryos^32^. Within this scenario, long term experiments on Zebrafish embryos can be performed to demonstrate the reliability of the system, verifying the absence of possible apoptosis processes.

## Results

**Figure 1:**
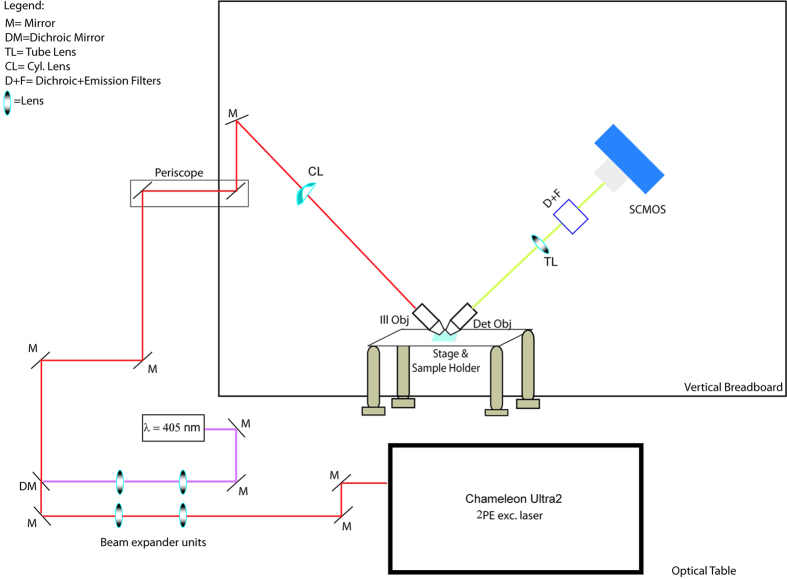
iSPIM architecture. The system is built on a vertical breadboard mounted on top of a standard optical table. The lasers (a 405 nm Coherent Obis solid state diode laser and a Chameleon Ultra 2 to provide two-photon excitation) send the light to two separate beam shaping units to provide comparable dimension of the beams. Mirrors and a periscope bring the laser beams to the vertical breadboard. The cylindrical lens creates the light sheets, which are again focused on the sample by the illumination objective. The detection objective collects the fluorescence and sends it to a tube lens, which focuses the light onto the chip of the SCMOS camera, while a dichroic mirror and a fluorescence filter select the correct wavelength for the detected light.

In this work we propose the use of a two-photon excitation light sheet based optical system for imaging biological tissues, such as mouse brain slices. The proposed architecture is based on an inverted selective plane illumination microscope as described in the section Optical set-up (Methods) and as shown in (Fig. 1).

The system is coupled with a tunable Ti-Sapphire IR laser thus allowing performing iSPIM imaging of biological tissues in the non-linear regime. Two photon excitation, thanks to the higher wavelengths used, is expected to improve the imaging capability compared to single photon excitation when scattering samples are imaged.

First, we characterized the excitation light sheet intensity distributions, to verify the comparability of the illumination profiles in terms of light sheet thickness in the linear and non-linear regime. However, because of the square dependence of two-photon excitation intensity, the FOV of 2P light sheet results to be relatively small compared to the single photon one. In order to compare the images acquired in both excitation regimes, only the region of uniformity of the excitation light sheets (the region within the 1/e^2 of the fitted curve can be considered as the uniform intensity area)^33^ has been taken into account as available FOV (251 ± 20 μm for two photon excitation and 947 ± 30 μm for single photon excitation). The thickness of illumination intensity distributions (measured in reflection mode, inserting a mirror at 45 degrees between the two objectives) found to be (2.4 ± 0.3) μm for visible light and (2.9 ± 0.3) μm for IR light.

The image frame rate and consequently the volumetric acquisition speeds, in terms of volumes per second, are limited by the integration time set on de CCD camera and mainly by a reasonable signal-to-noise ratio (SNR). For brain and zebrafish samples the exposure time has been set to 100 ms (0.04 Volumes per Second).

**Figure 2:**
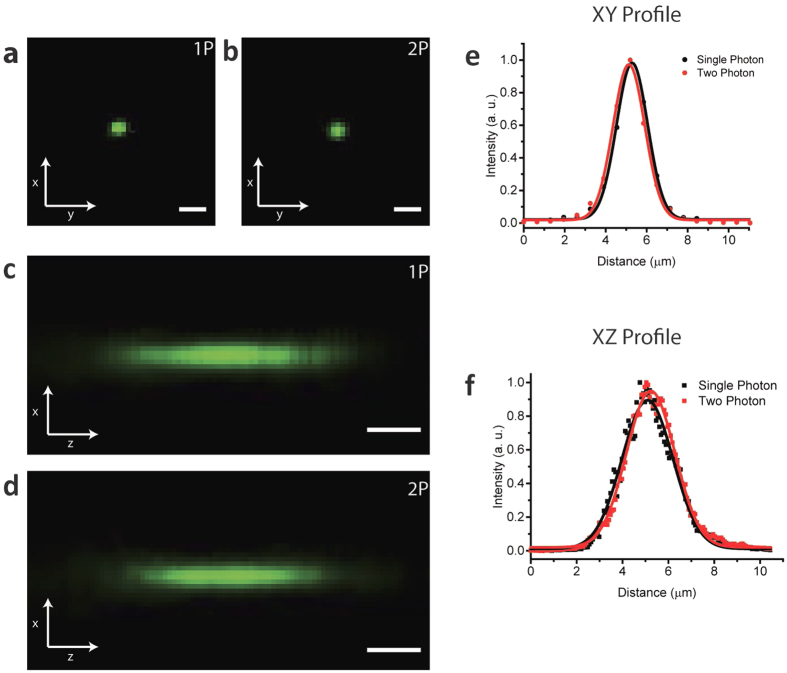
Comparison of the radial and axial PSF in both linear and nonlinear regimes. (**a,c**) Show respectively an example of the radial (**a**) and axial (**c**) intensity distribution of a 100 nm fluorescent bead excited with single photon excitation at 405 nm collected with a 10X, 0.3 NA. (**b,d**) Show respectively an example of the radial (**b**) and axial (**d**) intensity distribution of a 100 nm fluorescent bead excited with two-photon excitation at 740 nm collected with the same objective as in single photon case. (**e**) shows the linear profile of the intensity distribution shown in (**a,b**). The Full Width at Half Maximum (FWHM) of these profiles gives a direct estimate of the radial Point Spread Function of the system in 1P and 2P regimes (data shown in Table 1). Similarly (**f**) shows the axial profiles of the intensity distribution for single and two-photon excitation. Scale bars are 4 μm for (**a,b**) 1 μm (along z) for (**c,d**).

Imaging of a calibration sample (subresolved fluorescent beads) provides a direct estimation of the Point Spread Function (PSF) of the system, using the same objectives used for live imaging. Figure 2 shows the comparison of the PSFs in both excitation regimes and the values of the radial and axial resolution are shown in Table 1.

As expected, the lateral resolution in the non-linear regime is slightly poorer than the one in the single photon case^34^, while the axial resolution is equivalent and strongly related to the light sheet thickness of the light sheet.

Contrast capabilities and uniformity of the excitation intensity distribution in the iSPIM architecture are expected to be comparable to the ones obtained for a standard SPIM setup^27^. We focused our attention on investigating the imaging depth capabilities and contrast improvements provided by two-photon iSPIM when biological tissues, such as brain slices, are imaged.

**Figure 3:**
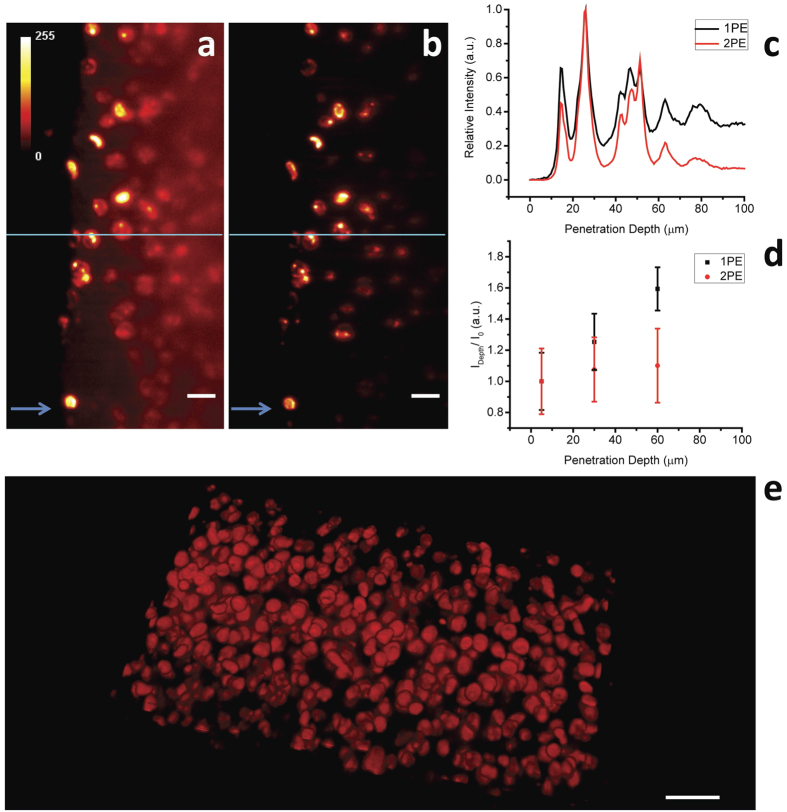
Quality evaluation of mouse brain slice imaging for single- and two-photon excitation: tissue imaging with 1PE (**a**) and 2PE (**b**) respectively. The arrows indicate the illumination direction of the light sheet. Each image was normalized to the maximum signal intensity. Scale bar 20 μm (**a,b**) 50 μm (**e**). (**c**) Line graphs showing normalized intensity profiles of 1PE (black line) and 2PE excitation (red line) along the blue lines in (**a**,**b**), respectively. (**d**) Ratio of the background intensities at a given depth over the intensity at 5 μm shows the background reduction provided by 2PE when deep imaging is performed. (**e**) 2PE volume rendering of brain slice reconstructed from the Z-stack (z-step 0.7 μm). A 3D reconstruction of mouse brain slice can be visible in Supplementary Movie 1. Excitation wavelength in 1PE experiment λ = 405 nm, I = 4.8 W/cm^2^, Excitation wavelength in 2PE experiment λ = 740 nm, I = 33 kW/cm^2^, illumination and detection objective Nikon CFI Plan Fluor 10X W, NA 0.3.

Imaging of 300 μm-thick coronal slices from mouse brain (Fig. 3), in which nuclei of neurons are labeled with Hoechst-33342, demonstrates that the proposed 2PE-iSPIM system is a suitable tool for 3D imaging of biological tissues. In particular, the comparison of single photon iSPIM (Fig. 3a) and two-photon iSPIM imaging (Fig. 3b) shows an improved image contrast.

It is evident that single-photon excitation shows a strong image blurring due to scattering effects and a consequent reduction of the signal to noise ratio (SNR) when the penetration depth increases. On the other hand, the relative intensity profile (Fig. 3c) shows the background signal reduction provided by two-photon iSPIM. A comparison of the relative background intensity ratio (defined as “C = I_depth_/I_0_”, where I_depth_ and I_0_ are the averaged background intensities at the given depth and at 5 μm from the brain slice surface, respectively) as a function of the depth demonstrates the improved performance, compared to the linear regime, for imaging depths up to 60 μm (Fig. 3d). Image stacks have been acquired at different depths, carefully checking the excitation light dose delivered to the sample in order to avoid photobleaching (intensity 33 kW/cm^2^) and a 3D rendering of the entire volume can be reconstructed in the non linear regime (Fig. 3e and Supplementary Movie 1). A 2D movie of the 3D stack is also shown in Supplementary Movie 2.

These experiments prove a remarkable 3D imaging capability in brain tissue, showing a strong background reduction compared to single photon excitation.

**Figure 4:**
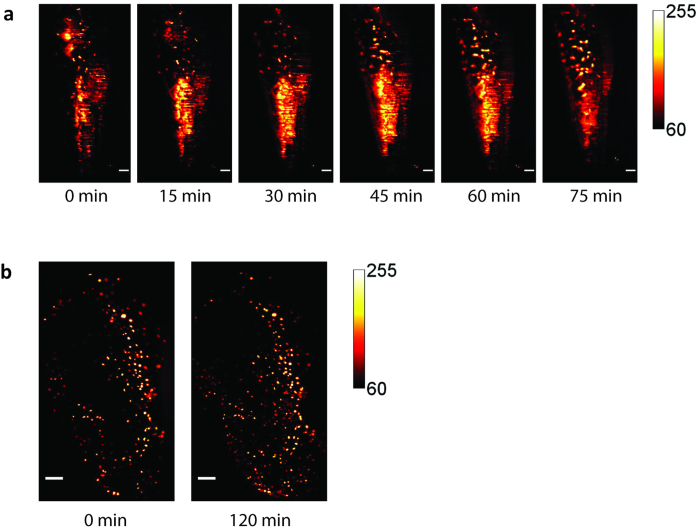
Time lapse acquisition of live dechorionated zebrafish embryo with two-photon excitation iSPIM. Panel (**a**) shows fluorescence temporal evolution of a 18 hpf embryo after staining with the vital dye 4-Di-2-Asp (DiAsp). Panel (**b**) shows temporal evolution of a 22 hpf embryo after staining with Hoechst 33342 (**b**). Scale bar 50 μm. Excitation wavelength λ = 720 nm for DiAsp (I = 20 kW/cm^2^), and 740 nm for Hoechst (I = 33 kW/cm^2^). Illumination and detection objective Nikon CFI Plan Fluor 10X W, NA 0.3. Exposure time 100 ms/frame (0.04 VpS).

Additionally, to test the reliability of the system for long term imaging of live samples, experiments were conducted on zebrafish embryos. As an example, to show the advantages provided by the system in terms of sample handling, easy buffer exchange and absence of sample gel embedding, we monitored the possible apoptosis processes in the embryo (Fig. 4).

In particular we labelled the zebrafish with DiAsp, an *in vivo* fluorescent dye, which allow visualizing the neuromasts, the surface mechanosensor organs of fish, in order to verify that the apoptosis process is neglectable overtime (Fig. 4a).

The reliability of the system for live long term imaging was further tested by marking nuclei with Hoechst 33342 to follow live developing fish over 120 mins, with almost negligible loss of fluorescence and photobleaching.

These data demonstrate that 2PE combined with an inverted SPIM arrangement represents a suitable imaging method for biological tissue imaging. In particular it represents a useful solution for extending the imaging performances of light sheet-based imaging to a wider range of scattering biological samples, such as mouse brain slices or retinal tissue or any other tissue usually incompatible with the gel-based embedding required in conventional SPIM.

## Discussion

This work proposes the use of an original optical system for 3D imaging of biological tissues based on the combination of an inverted SPIM architecture and two-photon excitation. From one side, the inverted architecture provides flexibility to the sample mounting procedure, getting rid of the gel-based sample preparation of conventional SPIM, and on the other side all the advantages brought by non-linear excitation in terms of reduction of the scattering effects and contrast improvement are exploited.

The characterization of the system is performed by imaging and calculating the Point Spread Function (PSF) both in a linear and non-linear excitation regime.

Imaging of thick biological tissues, such as hippocampal slices from mouse brain, proved as expected that 2PE allows for improved image quality and contrast while travelling in depth within the sample, with no need of complicate clearing procedures.

Moreover, experiments on live zebrafish proved that iSPIM three-dimensional imaging in the non-linear regime can be performed for long-term measurements, extending the application of this technique to a wider range of biological samples. We believe that the combination of two photon excitation with light sheet based architectures able to provide a flexible sample holding, such as iSPIM^12^,^13^,^14^ and the recently published single-objective SPIM^11^, represents a suitable platform for tissue imaging in general. In this way the advantages provided by two photon light sheet based microscopy can be extended to a larger variety of samples, such as for example brain slices or mouse retinas.

## Methods

### Optical set-up

The setup described here is an iSPIM custom built system, mounted on a vertically oriented optical breadboard (Fig. 1) suitable for both single (1P) and two-photon (2P) fluorescence excitation.

The microscope is equipped with 4 different laser lines (405, 488, 641 Coherent Obis Solid state diode lasers, and a 532 Coherent Sapphire) for single-photon excitation and multicolor imaging. Furthermore, to prime two-photon excitation, a Coherent Chameleon Ultra 2 (pulse width 140 fs across the wavelength tuning range, repetition rate 80 MHz) is also coupled to the system. A half-wave plate in front of a polarizing beam splitter (PBS) is used to tune laser intensity for two-photon excitation. Two independent beam-shaping units allow the tuning of the dimension of the Gaussian beams in order to create a comparable illumination distribution, in terms of light sheet thickness and available field of view (FOV), in the linear and non-linear regime. A 50 mm and a 150 mm focal distance lenses (Thorlabs AC254-050-A-ML and AC254-150-A-ML) are coupled to form a 3X telescope for visible light while a 50 mm and 200 mm focal distance (Thorlabs AC254-050-B-ML and AC254-200-B-ML) lenses are used to create a 4X telescope for 2PE light. This configuration allows for a comparable illumination condition for 1P/2P excitation.

The two laser paths are matched together with a dichroic mirror (Beamsplitter Q 720 SPXR Chroma) which reflects IR wavelength and transmits visible light.

The light-sheet is produced by a cylindrical lens (Thorlabs LJ1703RM) with a focal distance of 75 mm.

Due to the geometry of the system, the choice of objectives is limited by the working distance and the 90° orientation of the illumination and detection objectives. Thus, water immersion objectives with long working distance are required in order to minimize refractive index mismatch. Nikon CFI Plan Fluor 10XW, 0.3 NA, 3.5 mm WD has been chosen as the excitation objective. As a detection lens a Nikon CFI Plan Fluor 10X, 0.3NA, 3.5 mm WD has been selected to be a good compromise between NA and working distance.

The signal detected from the detection objective is focused by a tube lens (Thorlabs AC254-200-A-ML) onto a sCMOS Camera (Hamamatsu Orca Flash 4.0). Filters and dichroic mirrors (Chroma 420LP for 1P, Chroma D460/50 M, Semrock FF670 SDi 01 for 2P, Chroma SP680) allow for the selection of the fluorescence emission and for rejecting the excitation light.

The sample is mounted on a standard microscope petri dish (50 mm Sterilin Petri Dish, Thermo Scientific) filled with an aqueous medium.

A motor stage (Physik Instruments) allows coarse movement of the sample, while a piezo-driven stage (Physik Instruments P-563.3CD) allows 3D fine translation of the sample through the static light sheet (over 300 μm × 300 μm × 300 μm range).

Our implementation of the system provides a static light sheet trough through which the sample is translated in order to get a final 3D stack, as opposed to the previously published iSPIM architectures^12^,^14^, in which the light sheet was scanned through the static sample with a piezo moving the objective to maintain a constant focal plane.

A post-processing alignment procedure must be performed to reproduce a 3D stack because of the 45 degrees mismatch between the optical axis and the translation axis of the sample. A custom designed algorithm has been developed to align the images on the very same optical axis. Image J also provides plugins (Stackreg) to solve this issue^35^.

### Mice, brain sectioning and staining procedures

WT CD1 mice (Charles River) were used for this study. Animals were housed under standard conditions in the Animal Facility of “Fondazione Istituto Italiano di Tecnologia” (IIT). The IIT animal facility conforms to the standards of FELASA and is designated as a Research Facility by the Italian Ministry of Health (D.M. n. 29/2011-A 16/02/2011) and by authorization from the city of Genova. Animal health and comfort were veterinary-controlled, throughout lifetime of the project. All animal experiments, carried out in “accordance” with the approved guidelines, were performed in full compliance with the revised directive 2010/63/EU, Italian Legislation (art. 31 D.lgs.26/2014) and were approved by the Italian Ministry of Health (Authorization N° 214/2015-PR), and by the IIT-OPBA. Histology: Anesthetized animals (i.p. ketamine (90 mg/kg) and xylazine (5–7 mg/kg) were sacrificed by intracardiac perfusion with a solution of 4% paraformaldehyde (wt/vol) in phosphate-buffered saline. The brain was dissected out and immersed overnight in the same fixative at 4 °C. Prior to further processing brains were incubated in 30% sucrose-PBS solution (wt/vol). Frozen brains were cut to 300 μm sections using a HM-450 sliding microtome (Thermo). To reduce the possible auto fluorescence due to the paraformaldehyde, the brain slices were incubated in freshly prepared 1% sodium borohydride (NaBH_4_) in 0.1 M PBS (pH 7.4) for 30 min, before the staining procedure. Afterwards, slices were rinsed three times in PBS (0.1 M, pH 7.4), and pre-incubated in blocking solution consisting of 5% normal goat serum (NGS) and 0.2% Triton X-100 in 0.1 M PBS, pH 7.4 for 60 min at room temperature, to reduce non-specific staining. Cell nuclei were then stained with Hoechst-33342 (10 μg/ml in 0.1 M PBS, pH 7.4) (Invitrogen-Life Technologies) for 3 hours at room temperature, covered with aluminium foil to protect it from light. The stained brain slices were washed five times for 30 min each in PBS (0.1 M, pH 7.4) at room temperature, and maintained in PBS (as a mounting medium).

### Zebrafish and staining procedures

Wild-type (wt) adult zebrafish (*Danio rerio*) were purchased from a commercial source and were kept at 28 °C at 14 hr of light and 10 hr of dark per day. Fish were fed three times daily and were crossed as shown in the “Zebrafish Book”^36^. Fertilized eggs were chosen under a stereomicroscope (Stereo Discovery.V8, Zeiss Microscopy) and collected at 4 hours post fertilization (hpf). Healthy embryos were maintained in zebrafish embryo medium (i.e. NaCl, KCl, CaCl_2_.2H_2_O and MgCl_2_.6H_2_O; pH 7.3) and housed in an incubator at 28 °C.

Staining, Hoechst or DiAsp: For live staining, dechorionated zebrafish embryos at 18–22 hpf were incubated with 5 μg/ml of Hoechst (33342, Sigma) or 2 mg/ml of 4-(4-diethylaminostyryl)-N-methylpyridinium iodide (DiAsp, Sigma) in embryo medium for 15 min in the dark. Labeled embryos were rinsed three times with fresh embryo medium, immobilized on 0.5% agarose gel, and placed on a petri dish. The petri dish was filled with fresh embryo medium at 28 °C for imaging. All animal experiments were performed in full compliance with the revised directive 2010/63/EU, Italian Legislation (art. 31 D.lgs.26/2014) and the methods were carried out in “accordance” with the approved guidelines.

### Fluorescence beads calibration sample

Fluorescent microspheres with a diameter of 0.1 μm (TetraSpeck Microspheres, Invitrogen) were used to characterize the system and acquire the radial and axial PSF. A 1:10000 dilution in PBS was used to avoid creation of clusters. Excitation wavelength for 1P regime was 405 nm, while in 2P regime excitation wavelength was 740 nm (exposure time 10 ms/frame).

## Additional Information

**How to cite this article**: Lavagnino, Z. *et al.* 4D (x-y-z-t) imaging of thick biological samples by means of Two-Photon inverted Selective Plane Illumination Microscopy (2PE-iSPIM). *Sci. Rep.* **6**, 23923; doi: 10.1038/srep23923 (2016).

## Supplementary Material

Supplementary Video 1

Supplementary Video 2

Supplementary Information

## Figures and Tables

**Table 1:** Radial and axial Point Spread Functions of the system can be directly estimated, in single- and two-photon excitation regimes, measuring the Full Width at Half Maximum (FWHM) of the radial and axial profiles shown in Fig. 2.

Excitation regime	Wavelength nm	Objective	Radial (FWHM) μm	Axial (FWHM) μm
Single Photon excitation (1PE)	405	10X, 0.3 NA	1.78 ± 0.05	2.6 ± 0.1
Two Photon excitation (2PE)	740	10X, 0.3 NA	1.82 ± 0.04	2.5 ± 0.1

The average value of the FWHM of the radial profile in single photon excitation, statistically calculated over 20 measurements, was found to be 1.78 ± 0.05 μm while in Two-Photon Excitation was found to be 1.82 ± 0.04 μm. Similarly, the axial profiles of the intensity distribution for single and two-photon excitation have been calculated, showing average values of FWHM of 2.6 ± 0.1 and 2.5 ± 0.1 μm, respectively.
