# Supplementary material for: 4D (x-y-z-t) imaging of thick biological samples by means of Two-Photon inverted Selective Plane Illumination Microscopy (2PE-iSPIM)
Source: Sci Rep. 2016 Apr 1;6:23923. doi: 10.1038/srep23923 (PMC4817031; doi:10.1038/srep23923)
Supplement: Supplementary Information [file srep23923-s3.pdf]

Supplementary information:

4D (x-y-z-t) imaging of thick biological samples by means of Two-Photon inverted Selective Plane Illumination Microscopy (2PE-iSPIM).

Zeno Lavagnino[1*+], Giuseppe Sancataldo[1,2+], Marta d'Amora[1], Philipp Follert[3], Davide De Pietri Tonelli[3], Alberto Diaspro[1,4,5], Francesca Cella Zanacchi[1,4**].

Suplementary figure legend:

Supplementary Video 1 3D rendering of mouse brain slices. 3D rendering of the entire volume of brain slices can be reconstructed by iSPIM in the non-linear regime.

Supplementary Video 2 3D stack of mouse brain slices. 3D stack of the entire volume of brain slices imaged by iSPIM in the non-linear regime.
